# Unravelling risk selection in Spanish general government employee mutual funds: evidence from cancer hospitalizations in the public health network

**DOI:** 10.1007/s10198-024-01671-5

**Published:** 2024-02-20

**Authors:** Jaime Pinilla, Beatriz G. López-Valcárcel, Enrique Bernal-Delgado

**Affiliations:** 1 Department of Quantitative Methods for Economics and Management, Faculty of Economy, Business and Tourism, University of Las Palmas de Gran Canaria, Campus de Tafira, 34-35017 Las Palmas de Gran Canaria, Spain; 2 Data Science for Health Services and Policy Research Group, Aragon Health Sciences Institute, Institute for Health Sciences (IACS), San Juan Bosco 13 (CIBA Building), 50009 Zaragoza, Spain

**Keywords:** Risk selection, Private health insurance companies, Mutual Funds model, Exact matching, Spain, I13, I18

## Abstract

Government employees in Spain are covered by public Mutual Funds that purchase a uniform basket of benefits, equal to the ones served to the general population, from private companies. Companies apply as private bidders for a fixed per capita premium hardly adjusted by age. Our hypothesis is that this premium does not cover risks, and companies have incentives for risk selection, which are more visible in high-cost patients. We focus on a particularly costly disease, cancer, whose prevalence is similar among government employees and the general population. We compare hospitalisations in the public hospitals of the government employees that have chosen public provision and the general population. We analysed a database of hospital discharges in the Valencian Community from 2010 to 2015 (3 million episodes). Using exact matching and logistic models, we find significant risk selection; thus, in hospitalised government employees, the likelihood for a solid metastatic carcinoma and non-metastatic cancer to appear in the registry is 31% higher than in the general population. Lymphoma shows the highest odds ratio of 2.64. We found quantitatively important effects. This research provides indirect evidence of risk selection within Spanish Mutual Funds for government employees, prompting action to reduce incentives for such a practice. More research is needed to figure out if what we have observed with cancer patients occurs in other conditions.

## Introduction

Spain has two main statutory health systems. The most important one, funded in 1986, covers 95% of the population, is fully decentralised to the regions [namely, Autonomous Communities (AC)], and takes the form of a National Health Service (NHS), providing universal coverage for a comprehensive basket of benefits for virtually all Spanish residents. As an NHS, it is mainly funded by taxes; in addition to taxes, cost sharing (hardly 20% of the total expenditure in health in 2021) applies to outpatient pharmaceuticals and medical aids such as hearing aids and corrective lenses. Voluntary health insurance is bought by 14.6% of the total population, mainly as extra protection for services that are covered by public insurance but have long waiting lists or filters to access (family doctors, who are gatekeepers for hospital specialists) [1].

In the Spanish NHS resources and services are allocated and provided within administrative areas. The funding and purchasing mechanisms and the limited competition across areas entail no incentives for patients’ risk selection.

In addition, Spain has a second statutory health system established in 1975. State civil servants, armed forces workers and workers in the justice sector (hereinafter all of them referred to as government employees, GEs) are covered by Mutual Funds (MFs) that will manage, on behalf of the Social Security National Institute (INSS) health care benefits. After collecting funds from the Government and from the fees charged to GEs’ salaries, MFs will purchase services either to the public sector or to the private sector on the basis of a per capita premium. Irrespective of the type of provider GEs should get the same benefits as in the case of the general population.

Interestingly, on a yearly basis, GEs can opt either for public or private provision. In the first case, care is provided by the Regional Health Services who are in charge of the planning, purchasing and provision of services in the AC; in the case of private provision, they can choose among a number of private insurance companies. Periodically, in their role of purchasers, MFs (MUFACE, “Mutual Society of State Civil Servants”; MUJEJU, “General Judicial Mutual Society” and ISFAS, “Social Institute of the Armed Forces”) will establish purchasing agreements with the private health insurance companies, that, in this context, will play the role of services providers. Formally, it entails a public bid with companies willing to provide the services will be funded according to a per-capita premium.

In 2019, the system accounted for M€2230.7 (65% MUFACE, 31% ISFAS and 4% MUGEJU), overall 2.97% of the global expenditure on health, and covered (GEs (i.e., policyholders) and their relatives (namely, beneficiaries)) 2.2 million lives in 2020 (4.6% of the Spanish population).

**Fig. 1:**
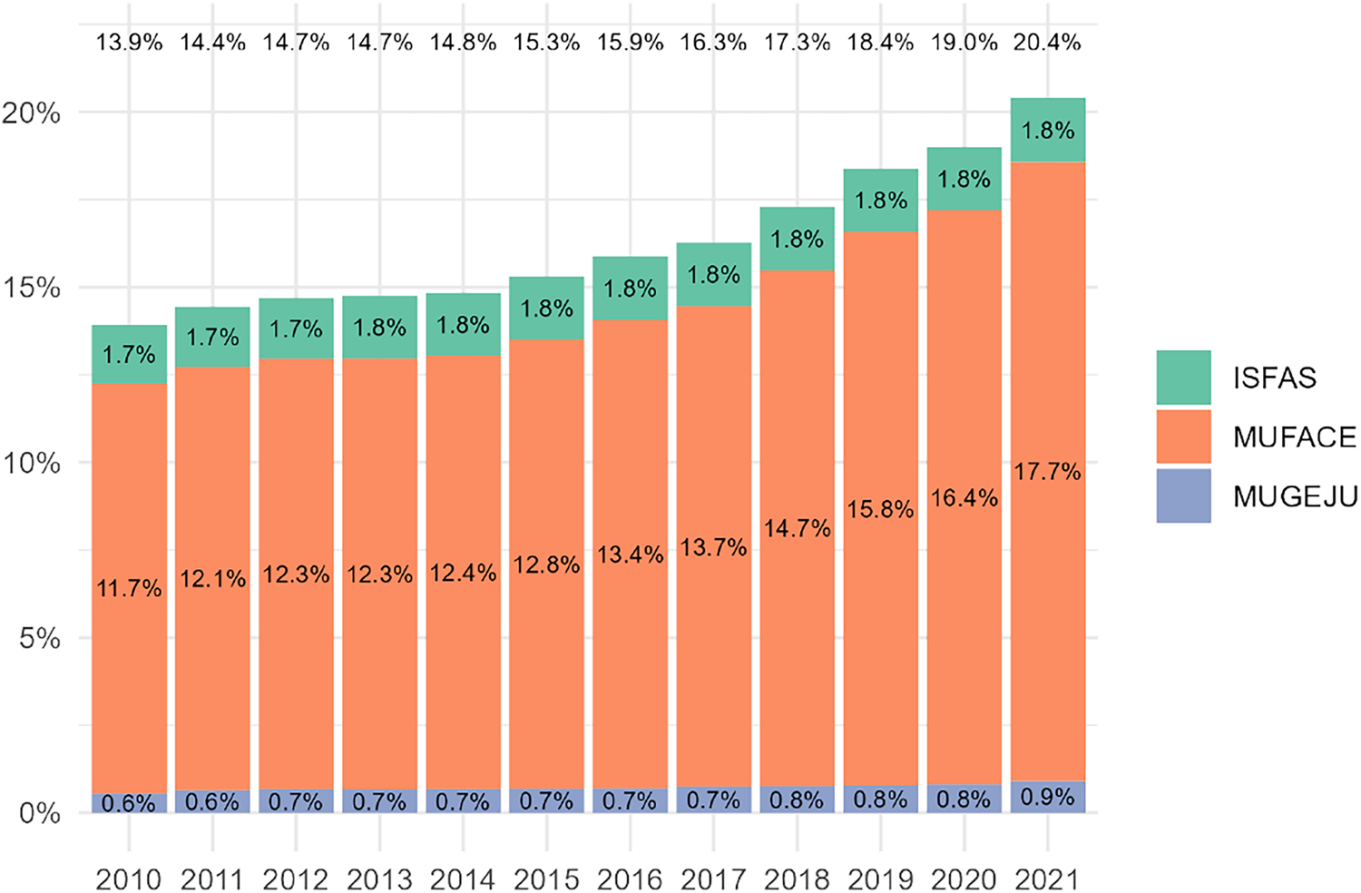
Evolution of the total number of government employees and relatives who opt for public provision from the National Health Service (2006–2021). Source: Created by authors based on the information available in the MUGEJU, MUFACE, and ISFAS reports [2–4]

Over recent years, the percentage of GEs and their relatives choosing the public provision has increased considerably; in 2010 it was only 13.9%; in 2021 around 20% opted for the public health coverage provided by the NHS. Figure 1 illustrates the dynamics of this choice using data from the memories of the three MFs.

Unlike, in the aforementioned NHS, where risk selection is unlikely, in the MFs system there is some room for risk selection—arguably, per capita premiums may not be high enough to cover high risks, not all the private companies have all the services to which policyholders and beneficiaries could access, justifying transferring patients to specialised public providers, and, finally, not all the private providers’ resources are allocated closed to the place of residence of the GE’s as in the case of the NHS.

In this article, we provide indirect evidence on the existence of risk selection by private companies covering GEs. We analyse public hospitals’ admissions due to cancer. We focus on cancer not as a prototypical representation of diseases but as a condition that is notably expensive once diagnosed [5]. The sample includes the universe of admissions both for the general population and for GEs who have opted for public provision.

Our paper is structured as follows: “Institutional background” provides an overview of the MF system, then we formulate the risk selection hypothesis according to the relevant literature and review some evidence for other countries. “Material and Methods” follows with a description of the data and the estimation procedures. “Results” presents empirical results, and finally, Sections “Discussion” and “Conclusion” conclude with a discussion of the policy implications of our findings.

### Institutional background

As said above, annually, GEs have the option to choose to be served by the public NHS providers or any of the private providers in those companies that sign the agreement with the MF for a period of time (currently, 2022–2024). Agreements are published in the Official State Gazette (in Spanish, Boletin Oficial del Estado) and detail the benefits and coverage (the same for all applicants). GEs can change public or private once a year, or at any time, subject to specific requirements. In either case, they are covered by the same basket of benefits. Private companies mainly use their network of private providers.

In this model of health insurance, purchasing and service provision, the private companies apply to a public bid against a prospective capitation payment adjusted for age intervals (see Table 1). Only a few private for-profit insurance companies sign the agreements. The other private companies operating in the market are not interested in participating, possibly because they estimate that their average healthcare costs will be higher than the financing agreed in the contracts.

**Table 1:** Comparison between MUFACE per capita payments to private companies and average healthcare expenditure in Spain, by age groups

Age group	(a) Capitation payment to private companies MUFACE 2023 (€ per month)	Capitation payment to private companies MUFACE Index	(b) Per capita average health expenditure in the NHS (general population) (€ per month))	Per capita average health expenditure in the NHS (general population. Index	Expenditure/capitation payment (b)/(a)
0−4	76.63	120.0	108	261.4	1.4
5−14	63.87	100.0	41	100.0	0.6
15−44	72.37	113.3	62	150.4	0.9
45−54	76.63	120.0	100	242.6	1.3
55−64	85.15	133.3	164.0	396.5	1.9
65−74	102.18	160.0	258.3	624.3	2.5
> 74	110.69	173.3	351.3	849.0	3.2

Sources: (a) Resolution of December 22, 2021, of MUFACE [6], (b) Blanco-Moreno A, De Domingo V [7]

(a) does not include medicines. Some specific high-cost services are excluded also and paid for separately. Base age group used for the Index was 5–14

Indeed, taking the case of MUFACE, Table 1 compares the average monthly expenditure per capita in the NHS (i.e., general population) with the per capita premium offered to private companies to cover MUFACE beneficiaries. Except for those aged 5–44 years, the payment to private insurance companies is notably lower than the cost of care provided by public network providers to the general population, and the gap increases with age from 45 years onwards. Between 45 and 54 years of age, the expenditure is 30% higher than the per capita premium, and from 75 years of age onwards, it is more than three times higher.

Although is adjusted for age, the prospective capitated system encourages risk selection because the private insurer's surplus is determined by the unit costs per insured and there are no contract restrictions, regardless of the existence of compensating mechanisms [8].

### Risk selection in private services under public financing

Risk selection is inherent in community-rated premiums or capitation payments without compensation mechanisms to reimburse companies’ high costs due to "bad" risks*.* Incentives for risk selection arise whenever there is competition combined with community-rated premiums (or at least significant constraints with respect to risk-rated premiums) [9]. Some international comparisons showcase risk selection in private services under public financing.

The choice between public and private insurance, which was in force in the Netherlands until 1986 and in Germany since 1970, led to the practice of risk selection: “Due to market failures in health insurance and differences in the regulatory frameworks governing public and private insurers, choice of public or private coverage creates strong incentives for private insurers to select risks and leads to risk segmentation, thereby breaching equity in funding health care, heightening the financial risk borne by public insurers and lowering incentives for private insurers to operate efficiently [10].

In Germany, public insurance (sickness funds), which is run by non-profit organisations and cannot exclude anyone, covers over 90% of the population. Private insurance premiums are risk-adjusted, while public ones are community premiums adjusted by income. Using microdata, the German Socio-Economic Panel Study for the period 2000–2007 [11], found risk selection favouring private insurers through the mechanism of switching from private to public health insurance after a negative health shock. Social funds do geographically-based risk selection (comparing low- and high-cost areas of health care provision). Thus, in competitive markets, health plans face incentives to exploit unpriced heterogeneity in risk [12].

Several countries utilise some type of risk equalisation scheme for essential insurance and enact laws requiring open enrolment (rejecting applications is forbidden), uniform insurance coverage, and co-payment limitations [13]. The equalisation fund, fed by contributions from the state and other agents, is managed by the regulator and oversees equalisation payments. These imply cross-subsidies among companies ranging from low risks to high risks. In countries with basic health insurance coverage with risk-adjusted premium subsidies, if risk adjustments are insufficient, sickness funds have financial incentives for risk selection, which may threaten solidarity, efficiency, quality of care and consumer satisfaction [14]. Several international studies look for evidence of risk selection in markets where insurance companies compete for good customers and regulation sets community or insufficiently risk-adjusted premiums, as was the case in the Netherlands [13]. With a rich two-year individual administrative database, including healthcare expenses and risk characteristics of more than 16 million individuals with basic health insurance, they find that the risk selection may threaten the quality of care for chronically ill people and may reduce the affordability and efficiency of healthcare. Sapelli and Vial [15] found risk selection in favour of private insurers in Chile. In Germany, the premium differential between public and private insurance affects the user who pays it. Healthy people who can choose have incentives to subscribe to private insurance; if they suffer a health shock, they have incentives to switch to a public insurer [10]. In the case of the MFs in Spain, given the intrinsic limitations of capitation financing, even adjusted by age, to equalise risk [16], and the arguably low per capita premiums (Table 1), once the contract is signed, participating private companies have the incentive to do risk selection throughout a number of mechanisms, thus attracting the maximum number of healthy GEs while dodging the maximum number of unhealthy or costly service-demanding GEs; setting up administrative barriers, either by relocating the places where services are provided or making marginal changes in the services provided; or by sending signals of low quality for specific high-cost treatments. Evidence on risk selection can be found in the Ombudsman report that states that [17] “private entities are ceasing to attend to MUFACE, MUGEJU and ISFAS mutualists”. This situation would affect mainly oncology and psychiatry specialties. In addition, evidence from the Central Independent and Civil Servants Union (CSIF), the most representative in the public administrations, demanded an urgent response after important cuts in medical oncology and radiotherapy in Madrid in the three insurance companies that had signed the agreement with MUFACE [18]. The media in Spain also echoed some patient complaints regarding these practices [19, 20]. Risk selection in the MF system may translate into higher costs in the NHS, as the NHS ends up acting as a “safety net” for those GEs dumped from private companies.

Our hypothesis is that in the MFs system Spain, where all GEs are covered by the MFs for a uniform basket of benefits, and companies apply for a fixed per capita premium hardly adjusted by age, private bidders will practise risk selection with oncologic patients. An over-incidence of cancer patients admitted to public hospitals from MFs, as compared to those from the general population, will be indirect evidence of risk selection.

## Materials and methods

A population-based observational retrospective study was conducted. The study period ranged from January 1, 2010, to December 31, 2015. The study covers all the public hospitals of the Valencian community (around 5 million population). We extracted the data required for the individual analysis from the episodes registered within the National Health System Hospital Discharge Records Database (CMBD); thus, all were publicly funded by the NHS. The CMBD database receives compulsory notification from around 98% of the public hospitals in Spain [21].

For each entry, we collected socio-demographic (sex, age, and municipality of residence), clinical data (date of admission, circumstances at discharge, principal diagnosis, and other concurrent clinical diagnoses, using the International Classification of Diseases, Ninth Revision, Clinical Modification (ICD 9 CM) [22]. Membership to MFs was identified. As mentioned, those belonging to MFs are state civil servants, members of the military or judiciary personnel who opted for the public provision. Our database has 2,988,283 total hospital admissions; out of them, 18,678 are MF-affiliated inpatients. From the study sample, we identified four cancer groups using two different definitions, as in two common classification índices—Charlson and Elixhauser [23, 24]. The comorbidity indices are used in medicine to gauge the overall severity of admission and to forecast hospital length of stay, costs, and in-hospital mortality. The anticipated hospital resource utilisation and death rate are higher with a higher score. The need for using both indices is the different specificity in the identification of cancer cases. We created four dummies: one for cancer as in Charlson [23]; the three remaining as in Elixhauser definitions, for metastatic carcinoma, solid tumour without metastasis, and haematological cancer (lymphoma) [24].

To provide evidence for the hypothesised over-incidence of MF-affiliated patients with cancer, we performed a two-stage analysis. We first pre-process the data using exact matching (EM) to match each MF inpatient with all inpatients from the general population with identical characteristics; thus, the treated are MF inpatients, and the "control" are non-MF inpatients. As the baseline characteristics of the patients are imbalanced between the groups, adjustments are needed. The exact matching was done using the MatchIt R package [25]. Specifically, we looked for a set of sociodemographic variables to perform exact matching. We matched the total hospital admissions based on four categorical variables: age, gender, year of admission, and municipality of residence.

After generating the matched dataset, regression is used to estimate the MF affiliation effect (treatment) on cancer hospitalisation (outcome). Note that we focus on a binary outcome and treatment, thus our method involves the estimation of the conditional odds ratio (OR) for the association, using logistic regression, and subsequently, the potential-outcome mean (POM), and the average treatment effect on the treated (ATT) [26]. In the logistic regression, each inpatient of MF has a unit weight and each one of his/her matched controls has a weight equal to 1/n, where n is the number of inpatients from the general population matched with him/her.

With the aim of assessing whether the likelihood for an MF affiliated to be admitted is different from the likelihood for the general population, then incurring in selection bias, we used data from the 2011 and 2017 waves of the Spanish National Health Survey (SNHS) [27]. Using a probit model, we studied the difference in the prevalence of long-term chronic health problems between MFs and the general population, adjusting for confounding variables like gender, age, household social class, and government employee status. The analysis incorporated individual sample weights from the NHS, ensuring national representativeness and consistent estimates.

## Results

Descriptive statistics for all variables before matching are provided in the “Appendix” for both collective GEs and the general population; see Table 4.

**Fig. 2:**
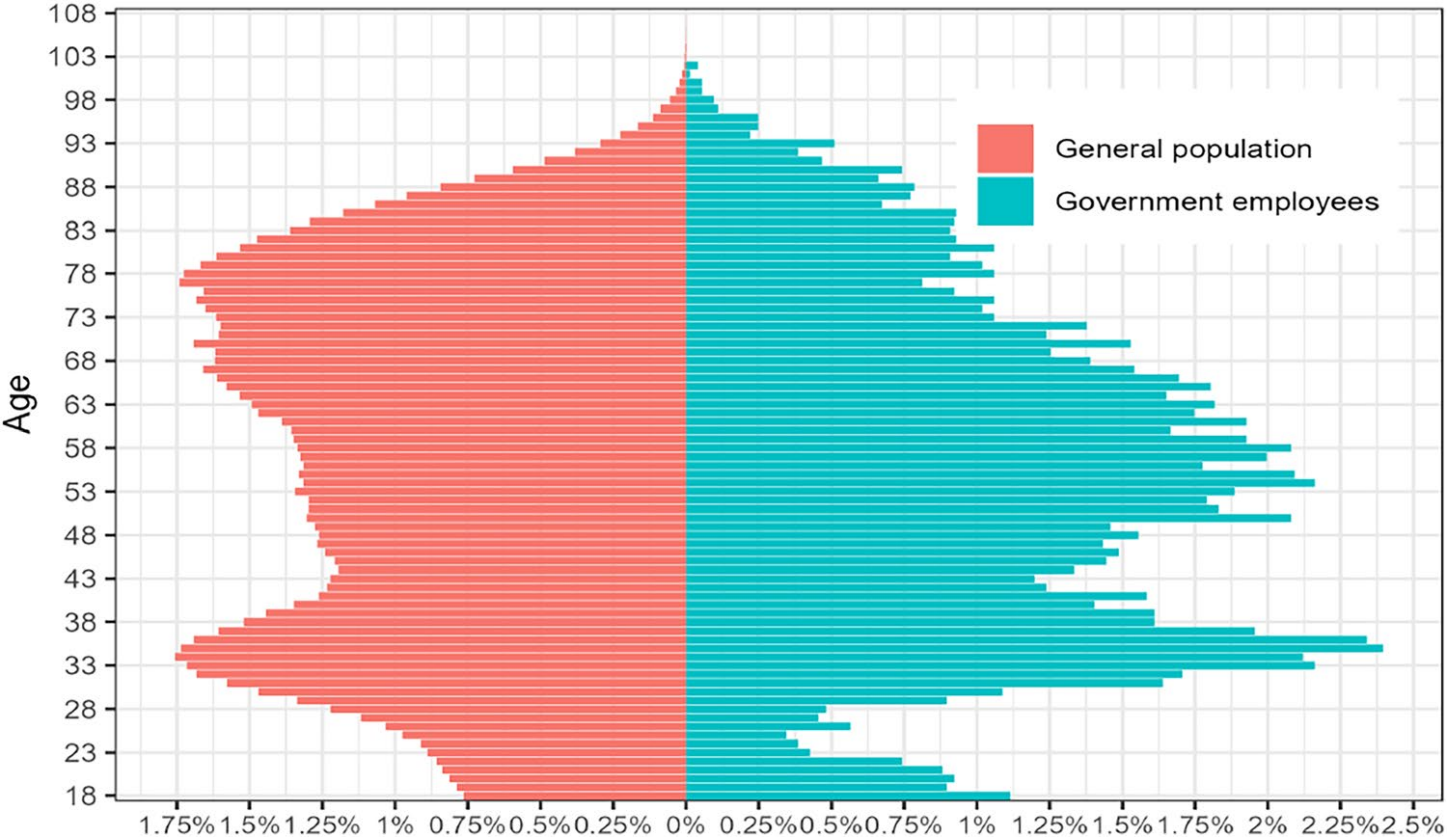
Distribution of hospital admissions by age group during the study period: government employees (Mutual Funds) opting for public provision and general population (National Health Service). Source: Created by authors; generated using data from the National Health System Hospital Discharge Records Database in Valencia Community 2010–2015

Figure 2 shows the age-sex structure for both groups in the population of the study. The wide base in the GEs collective indicates a higher proportion of hospital episodes in the categories 51–60 years and 61–70 years (37.28%). On the other hand, a wider top in the collective of the general population indicates a high proportion of episodes in the category of age 71 or over (37.18%).

Table 2 shows the result of the exact match. More than 96% of the individuals belonging to the group of interest (Mutual Funds’ affiliates) were matched. 74 observations were discarded due to the presence of missing values in some of the variables of interest.

**Table 2:** Summary for exact matching data

	Non-Mutual funds	Mutual funds	Total
All dataset	2,969,605	18,678	2,988,283
Matched	885,368	18,073	903,441
Unmatched	2,084,164	604	2,084,768
Discarded	73	1	74

Table 3 presents the estimated logistic regression results, odd ratios (OR), between MF inpatients and general population inpatients for the three models estimating the corresponding probabilities for each principal diagnosis considered.

**Table 3:** Regression adjustment results. Logistic models

Principal diagnosis	OR (std. err.)	ATT (std. err.)	POM (std. err.)	% of total admissions
Cancer^a^	1.3140*** (0.0424)	0.0282*** (0.0036)	0.1033*** (0.0012)	10.30%
Lymphoma^b^	2.6351*** (0.2895)	0.0118*** (0.0020)	0.0074*** (0.0003)	0.80%
Metastatic carcinoma^b^	1.3149*** (0.0712)	0.0092*** (0.0020)	0.0303*** (0.0005)	2.99%
Solid tumour without metastasis^b^	1.1341*** (0.0378)	0.1108*** (0.0031)	0.0921*** (0.0012)	9.11%
Number of observations 903,420				

Standard error in parentheses

*OR* odd ratio, *ATT* average treatment effects on the treated (ATT), *POM* potential-outcome means for general population inpatients

**p < 0.05; ***p < 0.01

^a^Defined as in Charlson comorbidities

^b^Defined as in Elixhauser comorbidities

All ORs are greater than unity and significant. Metastatic carcinoma has an OR = 1.3149, indicating that this condition is 31% more likely to be found in hospitalised GEs than in a hospitalised patient from the general population. For the other cancer conditions, non-metastatic solid tumours showed OR = 1.1341, other types of cancer, OR = 1.314, and lymphoma exhibited the highest OR = 2.6351. The cost of their treatment is in many cases higher than that of solid tumours [28]. ATTs are small in magnitude because cancer admissions are a small part, 10.3%, of total admissions. However, in relative terms ATTs indicate quantitatively important effects. The POM estimators are a way to gauge the average effect of the treatment, being a GE, on the probability of these cancer conditions being found within the hospital. According to the results in Table 3, the predicted POM for other types of cancer was 10.33% for the general population. This means that, if all the people in the sample belonged to the general population, we would expect a hospital incidence of this condition of 10.33%. The predicted POMs of hospitalisations with lymphoma, metastatic carcinoma, and solid tumour without metastasis among the general population were 0.74%, 3.03%, and 9.21%, respectively.

The results of the comparison between the general population and government employees in regard to chronic health conditions are detailed in Table 5 of the appendix. They revealed no significant difference in the reporting of chronic conditions between the cohorts. Our findings support the hypothesis that there is no inherent disparity in chronic disease prevalence between government employees and the general population. In the particular case of cancer, our estimation also finds no association between cancer diagnosis and MFs or non-MFs population. Showing a *p* value of 0.1684 on a total of 22,475 respondents, of which 1186 belonged to any MFs.

## Discussion

This study is the first one analysing population data covering all hospitalizations in public hospitals within a Spanish region (Comunidad Valenciana), where two population subgroups -general population and government employees—are using public hospitals in the treatment of cancer conditions.

According to economic theory, profit maximisation would encourage for-profit health insurance companies to practise risk selection when premiums do not capture the variety of risks. For the Spanish MFs model, we have shown evidence that specific oncological diagnoses are disproportionately more prevalent in those GEs hospitalised in a public hospital than in the general population, after adjusting for sociodemographic characteristics. As the prevalence of cancer in both collectives, GEs and the general population, do not differ significantly according to the Spanish National Health Survey, we conclude that our results may be indirect evidence of risk selection in the private companies covering the MF patients.

Pellisé [29] was the first to analyse mutualism, specifically MUFACE, as an example of managed competition in the Spanish healthcare system. She detected the presence of what she called "skim the cream", a type of the more general phenomenon of risk selection. It consists of making doubly insured members profitable: members of a private insurer who also have access to the public provider. People with double coverage would use private services only for minor, usually outpatient, health problems and public services for more serious and costly health problems.

What is the underlying mechanism of risk selection in the private companies serving the MFs? Private companies serving MFs have incentives to reduce their costs transferring high-risk GEs to the NHS as the per-capita-premium paid for the MFs is insufficiently risk-adjusted—see comparison in age-adjusted premium in Table 1 and reported evidence. Although they are legally obliged not to exclude anyone and to cover pre-existing conditions, in practice they use different mechanisms for risk selection, such as "being non-responsive" to the preferences of unhealthy people or sending subtle signals to GEs about access barriers or service quality. The aforementioned Ombudsman and civil servant unions reports provide evidence of this behaviour. [17, 18]. The consequence is that over the years more GEs are opting for public provision (see Fig. 1).

The MFs model is an example of competition between private, for-profit companies, for a defined population of GEs. From the perspective of public regulation, making it mandatory to compensate risks between private insurers through a system of reinsurance could, in principle, solve the problems of risk selection. However, the costs of regulation and the administrative costs of the companies themselves do not seem to support such a solution [30]. For this reason, it has received greater interest in the literature to initially try to neutralise possible selection risk problems by offering an adjustment in capitation funding that is capable of picking up the different degrees of risk incurred by the coverage of the different groups. Recommendations from Germany are "to avoid distorted competition between the two branches of health care financing, risk-adjusted transfers from private to public insurers should be instituted" [31]. Yet, the regulatory overheads and inherent administrative expenses of the companies often render this approach inefficient. Consequently, literature discourse has gravitated towards an alternative: refining capitation funding adjustments to effectively capture the varying risk levels associated with covering distinct groups.

With regard to the limitations, the optimal design to demonstrate risk selection in the MFs requires using longitudinal microdata from all GEs, with information on their choice of private companies and actual utilisation of services. Unfortunately, that information is not available. So, we designed a second-best test based on the comparison between the GEs that have chosen the public insurer and the general population treated in the public hospitals. Our approach has some strengths. We use registries covering virtually all admissions in public hospitals in a populated region of Spain from January 2010 to December 2015, including 3 million episodes of hospitalisation. We apply matching methods combined with logistic regression to estimate the counterfactuals; thus, the probability for a cancer condition to be present in a GE hospitalised in a public hospital as compared with a perfectly matched non-GE with the same age and sex and, hospitalised the same year and living in the same municipality. In the domains of economics, political science, epidemiology, and medical research, matching techniques have been used to reproduce a randomised experiment with nonrandomized data [32]. The most effective matching strategy is exact matching since it does not require any functional form assumption on either the treatment or outcome, and the covariate distributions are perfectly balanced. It is only possible when all the relevant variables for the matching are discrete or categorical (finite number of values or categories). However, a limitation of exact matching in practice is that often only a few units will remain after matching, so the estimated effect can be only generalised to a very limited population and can lack precision. Fortunately, we have a very large number of potential controls to match each government employee in our sample, so we did not have that problem. Anyway, matching methodologies exhibit limitations. We cannot be certain that matched respondents do not differ on unmeasured characteristics related to a respondent's likelihood to have a certain diagnosis or the underlying causes of choosing public provision. Although alternative matching methodologies exist to isolate a causal effect in nonexperimental settings (e.g. propensity score matching), we chose exact matching that optimises balance on pre-treatment covariates to reduce model dependence and potential bias in our outcomes of interest [33]. Finally, we may argue that the exact matching, although balanced, have not captured sufficiently the potential effect of other covariates not included, as comorbidities; the absence of a significant difference in the chronic conditions reported by GE vs the general population in the Spanish National Health Survey would, in any case, reinforce our hypothesis of risk selection.

A question remains on whether the indirect evidence of risk selection for patients with cancer conditions would be generalisable to other high-cost conditions. Further research should be required to confirm this hypothesis.

Our results may have important implications for policy-making in Spain. The MFs model, present since 1975, provides some privileges to GEs when it comes to choose when the focus is quicker access to specialised assistance and testing or more comfortable hospital stays; however, it could not represent a privilege for those with more severe conditions. (See the results of the question about “reasons for choosing between public and private” in the Spanish Health Barometer Survey [34], Table 6). However, the quality of services for complex processes, technology, and available means is still superior in the National Health Service, which may be the reason for government employees to choose the public provider, particularly those older GEs with chronic conditions or people who are risk-averse to serious illness.

Interestingly, there is a social debate on the advisability of generalising the MF model to the whole population, radically changing the healthcare system towards one based on the labour condition, no gatekeeping and free choice of provider, with an NHS in the background as a safety-net organisation. The underlying argument put forward by those who advocate this model is based on the “lower costs” of healthcare for GEs (as revealed by the capitation premium) as compared to the costs in the general population. However, as we have seen in this article, the differences, at least in cancer-related hospital admissions, seems to point definitively out to risk selection.

## Conclusions

Private companies serving the MFs model have incentives to attract healthier GEs while attempting to avoid treating individuals with higher healthcare needs. This behaviour potentially burdens the public health system, leading to an unjustified increase in public health spending.

Our study highlights the need for policy interventions to address risk selection within the MFs. By fostering a more balanced risk pool and preventing the shifting of costly patients to the public system. From a regulatory standpoint, to refine capitation funding in a way to effectively capture the varying risk levels associated with covering distinct groups could address risk selection concerns.

## Appendix

See Tables 4, 5 and 6.

**Table 4:** Descriptive statistics for the study variables

	Total sample				Matched sample^a^			
	Non-Mutual funds		Mutual funds		Non-Mutual funds		Mutual funds	
	Mean	N. obs	Mean	N. obs	Mean	N. obs	Mean	N. obs
Age in years (std. error)	59.93 (19.82)	2,969,596	57.16 (18.88)	18,678	57.21 (18.76)	885,368	57.21 (18.76)	18,073
Gender-female (1 yes; 0 no)	53.14%	2,969,541	55.09%	18,677	55.03%	885,368	55.03%	18,073
Diagnosed lymphoma (1 yes; 0 no)	0.80%	2,969,459	1.87%	18,678	0.74%	885,347	1.92%	18,073
Diagnosed solid tumour without metastasis (1 yes; 0 no)	9.10%	2,969,459	10.32%	18,678	9.21%	885,347	10.32%	18,073
Diagnosed cancer (1 yes; 0 no)	10.29%	2,969,459	13.07%	18,678	10.33%	885,347	13.14%	18,073
Diagnosed metastatic carcinoma (1 yes; 0 no)	2.98%	2,969,459	3.92%	18,678	3.03%	885,347	3.95%	18,073
Year of admission		2,969,605		18,678		885,368		18,073
2010	15.69%		13.89%		13.82%		13.82%	
2011	15.83%		15.04%		14.89%		14.89%	
2012	16.49%		15.31%		15.30%		15.30%	
2013	16.96%		17.50%		17.63%		17.63%	
2014	17.42%		18.81%		18.80%		18.80%	
2015	17.61%		19.46%		19.56%		19.56%	

^a^Weighted values to adjust for the imbalances in the sampling

**Table 5:** Probit models of reporting suffering from long-term chronic health problems: Spanish National Health Survey 2011 and 2017

	Spanish National Health Survey 2011	Spanish National Health Survey 2017	Spanish National Health Survey 2017
Dependent variable	Long-term chronic health problems	Long-term chronic health problems	Cancer condition diagnosed
Female (1 yes; 0 no)	0.1997 (0.0229)***	0.1980 (0.0228)***	0.1018 (0.0477)**
Age (years)	0.0300 (0.0007)***	0.0321 (0.0007)***	0.0188 (0.0011)***
Social class			
Class I (Ref.)			
Class II	0.0888 (0.0549)	0.0917 (0.0529)*	− 0.0843 (0.1147)
Class III	0.0972 (0.0436)**	0.1218 (0.0429)***	0.0046 (0.0941)
Class IV	0.1244 (0.0454)***	0.2162 (0.0459)***	0.0394 (0.0948)
Class V	0.1328 (0.0405)***	0.1965 (0.0399)***	− 0.0360 (0.0897)
Class VI	0.2014 (0.0477)***	0.1900 (0.0462)***	0.0176 (0.1009)
Government employee (1 yes; 0 no)	− 0.0929 (0.04770)	− 0.0273 (0.0513)	0.1684 (0.1124)
Number of observations	20,231	22,475	22,475

Social class based on the head of the household occupation

Class I: Directors and managers of establishments with 10 or more employees and professionals traditionally associated with bachelor's university degrees

Class II: Directors and managers of establishments with fewer than 10 employees, professionals traditionally associated with diploma university degrees and other technical support professionals. Athletes and artists

Class III: Intermediate occupations and self-employed workers

Class IV: Supervisors and workers in skilled technical occupations

Class V: Skilled primary sector workers and other semi-skilled workers

Class VI: Unskilled workers

****p* < 0.01; ***p* < 0.05; Linearized standard error in parentheses using the sample weights

**Table 6:** Spanish Health Barometer Survey 2015

Factor	Public (%)	Private (%)	Both (%)	No answer (%)
The technology and means available	68.8	21.9	8.5	0.8
The capabilities of the medical staff	63.8	15.9	19.5	0.8
The capabilities of the nursing staff	63.5	16.2	19.4	0.9
The speed with which you are attended	32.8	61.7	4.6	0.9
The information you receive	51.2	29.5	17.8	1.5
Personal treatment	47.4	35.6	15.8	1.2
The comfort of the facilities	39.1	51.4	8.1	1.4
Total number of responses 7746				

Question 7: I am going to read you a list of reasons why people might choose a public or a private health service. In your particular case, and always if you could choose, would you choose a public or private health service taking into account…?
